# Effects of Secondhand Smoke Exposure on the Health and Development of African American Premature Infants

**DOI:** 10.1155/2011/165687

**Published:** 2011-05-18

**Authors:** Jada Brooks, Diane Holditch-Davis, Mark A. Weaver, Margaret Shandor Miles, Stephen C. Engelke

**Affiliations:** ^1^Duke University School of Nursing, DUMC 3322, 307 Trent Drive, Durham, NC 27710, USA; ^2^Family Health International, 2224 E. NC Highway 54, Durham, NC 27713, USA; ^3^University of North Carolina at Chapel Hill School of Nursing, CB no. 7460, Chapel Hill, NC 27599, USA; ^4^Department of Pediatrics, Brody School of Medicine at East Carolina University, 600 Moye Boulevard, Greenville, NC 27834, USA

## Abstract

*Objective*. To explore the effects of secondhand smoke exposure on growth, health-related illness, and child development in rural African American premature infants through 24 months corrected age. *Method*. 171 premature infants (72 boys, 99 girls) of African American mothers with a mean birthweight of 1114 grams. Mothers reported on household smoking and infant health at 2, 6, 12, 18, and 24 months corrected age. Infant growth was measured at 6, 12, 18, and 24 months, and developmental assessments were conducted at 12 and 24 months. *Results*. Thirty percent of infants were exposed to secondhand smoke within their first 2 years of life. Secondhand smoke exposure was associated with poorer growth of head circumference and the development of otitis media at 2 months corrected age. Height, weight, wheezing, and child development were not related to secondhand smoke exposure. *Conclusion*. Exposure to secondhand smoke may negatively impact health of rural African American premature infants. Interventions targeted at reducing exposure could potentially improve infant outcomes.

## 1. Introduction

Premature infants are at greater risk for health and developmental problems than full-term infants, including asthma, otitis media, and growth failure as well as cognitive, language, and motor problems [1–3]. African American premature infants are at greater risk for these problems than other prematures [4], and rurality may further increase the risk for problems [5] because fewer rural children receive routine preventive care than children living in other areas [6]. Thus, identifying factors associated with outcomes is necessary to reduce the severity of health and developmental problems in the vulnerable population of rural African American premature infants. 

An environmental factor that may be associated with these poor outcomes is postnatal exposure to secondhand smoke, but to date the effects of secondhand smoke exposure on premature infants has received minimal attention. Premature infants are more likely to experience exposure to secondhand smoke because their mothers are more likely to smoke than mothers of full-term infants [7]. Therefore, the purpose of this secondary analysis was to explore the effects of postnatal secondhand smoke exposure on specific aspects of the health and development of rural African American premature infants.

Exposure to secondhand smoke may lead to growth problems in premature infants. The relationship between prenatal smoke exposure and intrauterine growth retardation, manifested as reduced weight, length, and head circumference at birth, is well documented in full-term infants [8]. Vielwerth et al. [9] reported a reduction in linear growth due to heavy prenatal smoke exposure; decreased third trimester growth velocity of weight, with lower birth weight and birth length, and increased growth velocity from birth to 6 months in weight and length. While prenatal smoke exposure is associated with initial fetal growth retardation [10], postnatal catch-up growth has been observed through 6 years of age [8]. However, the ongoing effects of postnatal smoke exposure were not considered in these studies. Only a few studies explored postnatal smoke exposure and growth, and no differences were reported in weight, length, and head circumference over time between children exposed to postnatal smoke and those not exposed [11]. Further, no study examined prematurely born children who are at greater risk for growth failure than full-terms [3] or focused on African Americans. 

Secondhand smoke exposure may also increase premature infants' risk of developing asthma and other related respiratory problems. Premature infants may be more sensitive to developing asthma due to their immature lung development [12], and prematurely born children are at higher risk for wheezing and asthma than children born at term [12, 13]. Studies have demonstrated associations between prenatal and/or postnatal smoke exposure to wheezing and asthma in childhood in full-term children [14]. Further, studies have reported that prenatal smoke exposure results in wheezing development in very low birthweight infants in early infancy [15]. In a sample of White, African American, and Hispanic very low birthweight infants (<1500 grams), infants exposed to secondhand smoke had a greater need for acute care for respiratory problems in the first year of life than infants who were not exposed [15]. Although evidence supports the relationship between prenatal smoke exposure and development of wheezing in infants and children, more research is needed to determine the association between postnatal secondhand smoke exposure and the development of wheezing in African American premature infants beyond the first year of life.

Exposure to secondhand smoke may also increase the risk of otitis media in premature infants. Otitis media with effusion (OME) and recurrent otitis media (ROM) are among the most commonly diagnosed illnesses of childhood [16]. Increased risk for recurrence of otitis media has been reported in prematurely born infants possibly due to the use of ventilatory assistance provided through nasotracheal and nasopharyngeal intubation, local immune system disturbance, and neuromotor impairment [1]. A number of studies have attempted to describe the relationship between postnatal exposure to secondhand smoke and otitis media; however, there has been little agreement between them. Some studies indicate a clear association between secondhand smoke exposure and otitis media [17]. In particular, heavy maternal smoking is associated with an increased risk for ROM in the first year of life for infants weighing less than 3500 grams at birth [18]. Other investigators have suggested that the occurrence of ear infections is not increased by postnatal exposure to secondhand smoke but is slightly increased by prenatal and combined smoke exposure [19]. Although a number of age groups have been included in these studies, they were typically older children, and the studies did not include premature infants or focus on African Americans. 

Finally, postnatal exposure to secondhand smoke may result in increased rates of cognitive and developmental problems in premature infants. Prematurely born children have high rates of developmental problems, including cognitive, language, and motor delays [2]. The extent to which household smoking might increase the risk of these problems is less certain. It has been suggested that prenatal smoke exposure results in reduced cognitive development [20] and poorer performance on language-related tasks [21]. However, the impact of postnatal smoke exposure remains unclear. Slykerman and colleagues found that exposure to maternal smoking during the first year of life was associated with increased risk of developmental delay in both full-terms and a subgroup of small for gestational age infants [22]. In contrast, others have found that postnatal smoke exposure did not significantly contribute to the risk of developmental delay in the first 2 years of life [23]. Few studies have examined cognitive and motor development of African American premature infants exposed to postnatal smoke following birth. Thus, the impact of postnatal exposure on development in premature infants remains unclear.

The purpose of this study, therefore, was to explore the effects of secondhand smoke exposure on the health and developmental outcomes of rural African American premature infants, who are at high risk for developmental delays and illnesses. Specifically, we determined the degree to which secondhand smoke exposure affected growth (weight, height, and head circumference), probability of illness (asthma and otitis media), and development (Bayley Mental Developmental Index and Psychomotor Developmental Index [24] and Preschool Language Scale-4 [25]) through 24 months corrected age. In these analyses, we controlled for other variables known to affect these outcomes. In the growth analyses, we controlled for the infant's size at birth, gender, SGA, and chronic lung disease [26–28]. In the analyses of the illness variables, we controlled for length of mechanical ventilation [4]. In all analyses, we controlled for maternal characteristics (receiving public assistance and education) [4].

## 2. Methods

This study was a secondary analysis of data obtained from a larger longitudinal randomized study (R01 NR05263) of a nursing support intervention for African American mothers of premature infants conducted from 2001 to 2007 [29, 30]. Since the outcome variables considered in this analysis were not affected by the intervention and smoking was not a focus of the intervention, participants from both the intervention and control groups were included in this report.

### 2.1. Participants

The participants in this study were African American premature infants who received neonatal care from one of two regional perinatal centers in the southeastern United States and their mothers. The infants were less than 35 weeks gestational age and considered high-risk for health and developmental problems because they either weighed less than 1,750 grams at birth or required mechanical ventilation. Infants were excluded if they had congenital neurological problems (such as Down Syndrome, congenital hydrocephalus, or microcephaly), were symptomatic from substance exposure, were hospitalized longer than 2 months post-term, were part of a higher-order multiple set, or were not in the custody of the biological mother. Their mothers lived in rural areas and small towns in a southeastern state. Mothers were excluded if followup for 2 years was unlikely (such as out-of-state residence) or if the family situation made asking for consent intrusive or affected the mother's ability to respond to the intervention (such as maternal HIV, maternal age less than 15, current diagnosis of major depression, or non-English speaking mothers). 

The sample for this analysis included 171 premature infants from the larger sample of 197 whose mothers provided data on whether or not smoking occurred in the household for at least one time point (2, 6, 12, 18, or 24 months corrected age). Eleven sets of twins were included in the sample. Seventy-two of the children (42.1%) were boys, and 99 (57.8%) were girls. Gestational age at birth for the infants ranged from 23.0 to 35.0 weeks (M = 28.4, SD = 2.8). Birthweight ranged from 340 to 2,110 grams including one infant large for gestational age (M = 1,114, SD = 386.8). Infant length at birth ranged from 25 to 45.5 centimeters (cm) (M = 36.9, SD = 4.5) and head circumference ranged from 19 to 36.5 cm (M = 25.7, SD = 3.0). Mechanical ventilation ranged from 0 to 163 days (M = 14.5, SD = 24) with 45 (26.3%) infants experiencing chronic lung disease. Maternal age ranged from 15 to 44 years (M = 26 years, SD = 6.5). One hundred ten (69%) of the mothers were not married, and they completed an average of 12.7 (SD = 1.8) years of education. One hundred twenty-five (78%) mothers reported receiving public assistance at some point during the study.

### 2.2. Measures

**Smoking**

At 2, 6, 12, 18, and 24 months corrected for prematurity, information on infant smoke exposure was collected from a maternally completed questionnaire about the infant's health problems since the last contact with the research team. Mothers were also asked to list everyone in the household who smoked.

**Infant Growth**

Three aspects of infant growth were measured at 6, 12, 18, and 24 months by a research assistant or clinical nurse: weight in kilograms using a calibrated scale, height in centimeters using a height board, and head circumference in centimeters using a tape measure.

**Common Illnesses**

The mother completed a brief health history on the infant at each follow-up contact (2, 6, 12, 18, and 24 months). Mothers were asked whether the infant had experienced asthma or wheezing and whether they had experienced an ear infection since the last contact. Infants whose mothers reported that the infant had experienced one of these problems were categorized into two groups at each time period, “wheezing” versus “no wheezing” and “otitis media” versus “no otitis media.”

**Child Development**

Trained psychologists assessed child development at 12 and 24 months corrected age using the Bayley Scales of Infant Development Second Edition (BSID-II) [24] and the Preschool Language Scale-4 (PLS-4) [25]. The BSID-II generates a Mental Development Index (MDI) and Psychomotor Development Index (PDI). The 12-month MDI was used to identify infants with severe cognitive problems, whereas the 24-month MDI was used to estimate a broad range of infant and toddler cognitive abilities (memory, habituation, problem solving, classification, language, and social skills) and visual-fine motor coordination. The PDI was used to measure motor abilities (gross and fine motor skills) that are independent of cognitive skills. Reliabilities have been reported as  .88 for the MDI and  .84 for the PDI [24]. The MDI was correlated at  .79 with the General Cognitive Index of the McCarthy Scales of Children's Abilities; the PDI was correlated at  .59 with the McCarthy Motor Scale [24]. The MDI was correlated with Full Scale IQ (*r* = .73), Verbal IQ (*r* = .73), and Performance IQ (*r* = .63) on the Wechsler Preschool and Primary Scale of Intelligence-Revised, which is only scored on older children [24].

Prelinguistic skills, social communication, and language skills were assessed using the PLS-4 [25], which is based on standardized scoring of about 1500 children aged 2 weeks to 6 years, including children with disabilities, and 39.1% of whom were minorities [25]. The PLS-4 is standardized, such that mean at each age is 100 and standard deviation is 15. The scale is administered in 15 to 40 minutes. The PLS-4 has been reported to have good reliability and validity [25]. Estimated internal consistency ranged from  .81 to  .79 [25]. With good construct and discriminant validity, the PLS-4's concurrent validity was assessed by comparing scores with other measures of language skills and resulted in high correlations: Denver II correlated with normal scores falling within 1.0 standard deviations of the mean on the PLS-4; the Auditory Comprehension scores on the PLS-4 (an earlier version of the scale) and the PLS-4 were correlated  .65 and the Expressive Communication scores were correlated  .79 [25].

**Infant Neonatal Medical Data**

The infants' medical records were reviewed during hospitalization for descriptive data on infant gender and infant characteristics (such as gestational size) and neonatal illness severity (such as length of mechanical ventilation in days and chronic lung disease).

**Demographic Characteristics**

Demographic information was collected at enrollment in the hospital and was updated at the 2-, 6-, 12-, 18-, and 24-month contacts. Maternal report of years of completed education and family use of public assistance were used to measure socioeconomic status (SES). Other demographic information collected from the mother included gender and age of the child, maternal age, race, occupation, marital status, spouse, and head of household.

### 2.3. Procedures

The original study was approved by the Institutional Review Boards for protection of human subjects of the participating institutions. Mothers provided informed consent for their and their infants' participation when the infants were no longer critically ill (not receiving mechanically ventilation and not with an immediately life-threatening medical condition) as long as an additional hospital stay of at least 1 week was anticipated. Infants and mothers were followed in the hospital and after discharge until 24 months corrected age. All ages used are corrected for prematurity. Data points used in this report were 2 (mail contact), 6 (home visit), 12 (clinic visit), 18 (home), and 24 months corrected age (clinic visit). Mothers were compensated each time they completed questionnaires (at enrollment in the hospital and 2, 6, 12, 18, and 24 months) and reimbursed for travel and paid for incidental expenses (lunch, parking, etc.) during the visits to the clinic for developmental assessments at 12 and 24 months. The infant was given a small gift at each home visit.

### 2.4. Data Analysis

An alpha level of.05 was used to establish significance for two-tailed tests. All data were first analyzed using any smoker in the home as the predictor and then repeated using maternal smoking as the predictor. General linear mixed modeling was used to examine the relationship of smoking in the household to infant growth (weight, height, and head circumference at 6, 12, 18, and 24 months) and cognitive and motor development (MDI at 12 and 24 months and PDI at 12 and 24 months) longitudinally. The general linear mixed model (hereafter referred to as mixed model) is a flexible statistical procedure for analyzing continuous longitudinal data that accommodates missing values and mistimed data [31]. However, *R* ^2^ cannot be determined in an unambiguous manner for the general linear mixed model because the model has correlated errors and multiple variance terms, not just a single one. Parameters of the mixed model include population (fixed) effects and individual (random) effects. With this approach, each subject's repeated measures on the BSID-II and growth were first parameterized as an individual growth trajectory plus an error term. The estimated trajectories were then modeled as a function of differences between individuals on each of the independent variables. Because the ages of the children at the contacts varied slightly, the actual age of the child in weeks past term (40 weeks postmenstrual age) was used in analyses. Only the intercept and age were included in the random effects component, providing mother-child dyad-specific intercepts and slopes across age. The models also include random family effects to account for correlation between observations for twins. Covariates for growth outcomes included child characteristics (gender; weight, length, or head circumference at birth; being small for gestational age), child illness severity (chronic lung disease), and maternal characteristics (public assistance, maternal education). Covariates for cognitive and motor development (MDI at 12 and 24 months and PDI at 12 and 24 months) included maternal characteristics (public assistance and maternal education).

A linear mixed model was also used to examine the relationship of smoking in the household to PLS-4 (language) scores. However, because the data were only collected at one time point, this model only included a random family effect. Covariates included maternal characteristics (public assistance and maternal education).

The likelihoods of having wheezing or asthma and otitis media were analyzed using generalized estimating equations (GEE) [32] with a logistic regression model. GEE can also handle missing and mistimed values, as occurred in this study. Covariates included maternal education, public assistance, and length of mechanical ventilation.

## 3. Results

Exposure to secondhand smoke varied over the first 2 years of life: 13% of infants were exposed at 2 months, 20% at 6 months, 21% at 12 months, 27% at 18 months, and 33% at 24 months corrected age. Thirty percent of infants experienced secondhand smoke exposure within their first 2 years of life. Mothers with less education were more likely to smoke at each visit (Spearman's correlation ranged from about −0.17 at 2 months to about −0.24 at 6 months). There is no evidence that the intervention affected infants' exposure to secondhand smoke. Thirty-three percent of infants in the control group were exposed to secondhand smoke compared to 28% of infants in the intervention group (*P* = .452). In addition, 20% of the infants in the control group versus 23% of infants in the intervention group were exposed to maternal secondhand smoke (*P* = .639). The raw, unadjusted means for infant growth, illness, and developmental outcomes by household smoke exposure status and age are shown in Table 1.

### 3.1. Effects of Secondhand Smoke Exposure on Infant Growth

The general linear mixed models for infant growth outcomes are shown in Table 2. There was no main effect of household smoke exposure or interactions involving exposure to household smoking on weight and height. After 6 months, the head circumferences of infants exposed to household smoking showed slower growth than those of nonexposed infants. When the growth analyses were repeated using maternal smoking, rather than exposure to any household smoking, the findings were the same for head circumference. 

### 3.2. Effects of Secondhand Smoke Exposure on Health Problems

GEE results for childhood illness outcomes are shown in Table 3. The only significant effect for otitis media was that infants exposed to maternal smoking had 5.5 times the odds of otitis media at 2 months corrected age than non-exposed infants. The development of wheezing was not related to exposure to household smoking.

### 3.3. Effects of Secondhand Smoke Exposure on Child Development

The effects of household smoking on child development are shown in Table 4. Mixed general linear models provided no evidence that the MDI and PDI differed between infants with exposure to household smoking and non-exposed infants. Generalized linear modeling provided no evidence that infants with exposure to household smoking differed from non-exposed infants in language skills at 24 months. The results were the same when the models were calculated using maternal smoking.

## 4. Discussion

One-third of our sample of rural African American premature infants experienced postnatal exposure to secondhand smoke by 24 months. Passive exposure to secondhand smoke in the first 2 years of life has been reported to be as low as 17% [33] and as high as 75% in some European countries, such as Poland [34], and secondhand smoke exposure in a rural US children 16 years of age and younger was 83% [35]. Because very few studies have examined secondhand smoke exposure in rural premature children, whether our findings about secondhand smoke exposure in African American premature infants are greater than expected is difficult to determine. 

Maternal smoking during the postnatal period was highly correlated with lower maternal education. Similarly, Ey and colleagues reported that mothers with less than 12 years of education (30%) were more likely to smoke than mothers who received 13 to 16 years of education (26%) or mothers with more than 16 years of education (11%) [18]. In a study that included premature infants, being African American, having a parent with less than a college education, and living in a low-income home resulted in the highest exposure to secondhand smoke [36]. In the current study, mothers averaged less than 13 years of education and were likely to require public assistance, suggesting that their infants may be at increased risk for exposure to secondhand smoke. 

Similar to the results of other studies [8, 9, 11, 37–39], we found that African American premature infants exposed to secondhand smoke experienced slower growth in head circumference than non-exposed infants through 24 months corrected age. In this study, the differences in head circumference size between the exposed and non-exposed infants remained significant through 24 months corrected age. These findings are consistent with size differences that have been reported between children whose mothers smoked during pregnancy and those whose mothers did not smoke until 2 years of age [11] or later [8, 39]. Because exposure to prenatal smoking data was not assessed in this study, it is impossible to identify the exact cause of the slower postnatal growth. Although the possibility of effects from prenatal exposure to smoking cannot be eliminated, exposure to secondhand smoke during the postneonatal period appears to exert negative effects on growth in infant head circumference. This finding is concerning because poor head circumference growth has been related to poorer health and developmental outcomes [40–44]. Thus, premature infants exposed to postnatal secondhand smoke might show problematic outcomes after 24 months. Additional studies examining premature infants' exposure to both prenatal and postnatal smoke are needed to determine the individual and combined effects of prenatal and postnatal secondhand smoke exposure on growth.

Unlike other studies [14, 45, 46], we did not find an increased risk for wheezing in children exposed to secondhand smoke. Several explanations may account for this difference. First, the lack of an effect may have been because smoking exposure has only a small effect on wheezing risk at this age and the study lacked the power to detect a small effect. The impact of secondhand smoke exposure may not be as large in prematurely born children who are already at increased risk of wheezing and asthma. In addition, another explanation is that the increased risk found in other studies may be a combined effect of prenatal smoking and secondhand smoke exposure during childhood. Of the few studies including rural children, none found any evidence that rural residence and exposure to secondhand smoke were associated with wheezing and asthma [35, 47, 48]. Research including multiethnic samples of premature infants living in rural and urban areas may be beneficial in determining the role of residence on asthma and wheezing incidence. 

However, we did find a greater likelihood for otitis media at 2 months corrected age if the mother smoked. This increased likelihood is consistent with findings in other studies [17, 18, 49] during the first year of life. Still others have failed to establish a positive association between maternal smoking and otitis media [50–52]. However, most of these studies examined otitis media in children over the age of 1 year, and we did not find a significantly increased risk of otitis media after 2 months. In addition, no studies examined for otitis media in rural children. Therefore, only limited studies have been conducted to determine the impact of secondhand smoke exposure on otitis media in premature infants living in rural settings and more are needed.

Like some investigators [23], we found no evidence of differences in mental, psychomotor, and language development between infants exposed to secondhand smoke and non-exposed infants in the first 2 years of life. Similar findings have been reported for a subgroup of infants who were small for gestational age. In these infants, the association between maternal smoking during the first year of life and developmental delay was not significant [22]. This suggests that the point of exposure (e.g., during the prenatal period) may be an important factor in determining the degree to which early child development is affected. Lack of data on prenatal smoking in this study makes it difficult to determine the relative effects of prenatal smoking and those of secondhand smoke exposure during the postnatal period on child development. Also, the premature infants in our study had minimal smoking exposure in the third trimester because of their hospitalization in a neonatal intensive care unit. More studies examining cognitive and motor development of rural premature infants exposed to prenatal and postnatal smoke in early childhood are warranted.

## 5. Conclusions

In conclusion, factors in the immediate social environment, particularly secondhand smoke exposure, may negatively impact health outcomes of premature children who are at high-risk for negative outcomes because of their decreased birthweight and compromised immunity. Because a significant number of infants in this study were exposed to secondhand smoke by 24 months corrected age, health care interventions targeted at reducing secondhand smoke exposure could potentially minimize its influence on the growth and health outcomes of rural, African American premature infants. Our results reveal that secondhand smoke exposure is associated with poorer infant growth and the development of otitis media. However, our study only continued until 24 months corrected age. Thus, we do not know whether exposure to secondhand smoke continuing to later ages would lead to additional health problems, but it is likely that the health consequences of secondhand smoke exposure would continue to increase with increasing age. Health providers working with rural prematurely born African American children exposed to secondhand smoke need to assess for its effects on growth, development, and illness; provide appropriate treatment and follow-up care; educate parents about associated risks and strategies to reduce exposure in the home; and provide smoking cessation interventions and assistance to parents. 

## Figures and Tables

**Table 1:** Raw, unadjusted means for infant growth, illness, and developmental outcomes by household smoke exposure status and age.

Variables	Mean				
	2 mo. (*n*)	6 mo. (*n*)	12 mo. (*n*)	18 mo. (*n*)	24 mo. (*n*)
*Height *(cm)					
Exposed		65.4 (23)	74.0 (28)	81.6 (22)	85.8 (42)
Non-exposed		65.7 (89)	73.7 (111)	80.9 (72)	85.6 (82)
*Weight *(kg)					
Exposed		7.8 (26)	9.4 (28)	11.5 (27)	12.0 (44)
Non-exposed		7.7 (94)	9.2 (114)	10.9 (81)	11.8 (85)
*Head circumference *(cm)					
Exposed		42.8 (19)	45.4 (27)	47.6 (24)	48.0 (44)
Non-exposed		42.8 (84)	45.5 (112)	46.9 (69)	47.8 (85)
Wheezing*					
Exposed	0.2 (20)	0.3 (29)	0.4 (34)	0.4 (34)	0.4 (46)
Non-exposed	0.2 (134)	0.4 (112)	0.5 (123)	0.4 (92)	0.5 (93)
Otitis media*					
Exposed	0.2 (20)	0.4 (29)	0.5 (33)	0.5 (34)	0.4 (46)
Non-exposed	0.1 (134)	0.3 (112)	0.5 (124)	0.4 (92)	0.4 (92)
*MDI*					
Exposed			90.5 (32)		75.5 (44)
Non-exposed			92.6 (114)		79.4 (91)
*PDI*					
Exposed			83.0 (32)		82.6 (44)
Non-exposed			87.9 (113)		88.5 (91)

* indicates the proportion with the event.

**Table 2:** Effects of secondhand smoke exposure and covariates from mixed model analyses for infant growth outcomes.

Variables	Estimate (SE)	DF	*t* value	pr > |*t*|
*Weight*				
Household smoker versus no smoker				
Mean change from 6 to 12 months	−0.03 (0.19)	321	−0.14	0.8859
Mean change from 6 to 18 months	0.03 (0.26)	311	0.10	0.9199
Mean change from 6 to 24 months	0.16 (0.27)	211	0.59	0.5576
Maternal smoker versus not				
Mean change from 6 to 12 months	0.05 (0.21)	318	0.24	0.8134
Mean change from 6 to 18 months	0.13 (0.29)	298	0.46	0.6426
Mean change from 6 to 24 months	0.25 (0.29)	191	0.85	0.3947
*Height*				
Household smoker versus no smoker				
Mean change from 6 to 12 months	0.67 (0.79)	310	0.85	0.3937
Mean change from 6 to 18 months	1.04 (1.04)	306	1.00	0.3166
Mean change from 6 to 24 months	1.12 (0.95)	205	1.18	0.2398
Maternal smoker versus not				
Mean change from 6 to 12 months	0.80 (0.91)	308	0.88	0.3792
Mean change from 6 to 18 months	1.12 (1.21)	297	0.93	0.3552
Mean change from 6 to 24 months	0.96 (1.11)	193	0.86	0.3894
*Head circumference*				
Household smoker versus no smoker				
Mean change from 6 to 12 months	−1.18 (0.36)*	289	−3.31	0.0011
Mean change from 6 to 18 months	−1.58 (0.49)*	273	−3.24	0.0014
Mean change from 6 to 24 months	−1.20 (0.45)*	189	−2.63	0.0092
Maternal smoker versus not				
Mean change from 6 to 12 months	−1.29 (0.44)*	289	−2.93	0.0036
Mean change from 6 to 18 months	−1.76 (0.61)*	269	−2.87	0.0044
Mean change from 6 to 24 months	−1.39 (0.58)*	193	−2.41	0.0171

*indicates that *P* values are significant at the  .05 level.

**Table 3:** Effects of secondhand smoke exposure from GEE analyses for infant health problems.

Variables	OR estimate (SE)	Confidence limits
*Health*		
Wheezing		
Household smoker versus no smoker at 2 months	0.86 (0.56)	0.24, 3.11
Household smoker versus no smoker at 6 months	0.71 (0.33)	0.29, 1.77
Household smoker versus no smoker at 12 months	0.52 (0.20)	0.24, 1.12
Household smoker versus no smoker at 18 months	0.82 (0.33)	0.37, 1.79
Household smoker versus no smoker at 24 months	0.74 (0.26)	0.37, 1.47
Maternal smoker versus no maternal smoker at 2 months	1.19 (0.84)	0.30, 4.76
Maternal smoker versus no maternal smoker at 6 months	0.71 (0.41)	0.23, 2.19
Maternal smoker versus no maternal smoker at 12 months	0.66 (0.31)	0.26, 1.66
Maternal smoker versus no maternal smoker at 18 months	0.95 (0.44)	0.39, 2.34
Maternal smoker versus no maternal smoker at 24 months	0.68 (0.28)	0.30, 1.53
Otitis media		
Household smoker versus no smoker at 2 months	3.06 (2.30)	0.70, 13.33
Household smoker versus no smoker at 6 months	1.25 (0.51)	0.56, 2.78
Household smoker versus no smoker at 12 months	0.85 (0.31)	0.42, 1.74
Household smoker versus no smoker at 18 months	1.58 (0.58)	0.77, 3.23
Household smoker versus no smoker at 24 months	0.96 (0.36)	0.47, 2.00
Maternal smoker versus no maternal smoker at 2 months	5.53 (3.98)*	1.35, 22.68
Maternal smoker versus no maternal smoker at 6 months	1.45 (0.71)	0.56, 3.77
Maternal smoker versus no maternal smoker at 12 months	0.64 (0.28)	0.27, 1.53
Maternal smoker versus no maternal smoker at 18 months	0.95 (0.40)	0.41, 2.18
Maternal smoker versus no maternal smoker at 24 months	0.78 (0.33)	0.34, 1.78

* indicates that *P* values are significant at the  .05 level.

**Table 4:** Effects of secondhand smoke exposure and covariates from mixed model analyses for Bayley MDI and PDI and the general linear model for PLS-4.

Variables	Estimate (SE)	DF	*t* value	pr > |*t*|
*Development*				
MDI				
Household smoker versus no smoker				
Mean change from 12 to 24 months	−0.34 (3.01)	149	−0.11	0.9099
Maternal smoker versus no maternal smoker				
Mean change from 12 to 24 months	−3.49 (3.52)	153	−0.99	0.3238
PDI				
Household smoker versus no smoker				
Mean change from 12 to 24 months	−0.92 (5.82)	149	−0.16	0.8745
Maternal smoker versus no maternal smoker				
Mean change from 12 to 24 months	−7.72 (6.93)	149	−1.12	0.2665
PLS-4				
Household smoker versus no smoker at 24 months	−3.15 (2.49)	120	−1.26	0.2086
Maternal smoker versus no maternal smoker at 24 Months	−1.22 (2.79)	120	−0.44	0.6633

*indicates that *P* values are significant at the  .05 level.
